# The State of Evaluation Research on Food Policies to Reduce Obesity and Diabetes Among Adults in the United States, 2000–2011

**DOI:** 10.5888/pcd12.150237

**Published:** 2015-10-29

**Authors:** Nicholas Freudenberg, Emily Franzosa, Nancy Sohler, Rui Li, Heather Devlin, Jeanine Albu

**Affiliations:** Author Affiliations: Emily Fransoza, Nancy Sohler, City University of New York School of Public Health, New York, New York; Rui Li, Heather Devlin, Centers for Disease Control and Prevention, Atlanta, Georgia; Jeanine Albu, St Luke’s Roosevelt Hospital Center, Obesity Research Center, New York, New York.

## Abstract

**Introduction:**

Improvements in diet can prevent obesity and type 2 diabetes. Although policy changes provide a foundation for improvement at the population level, evidence for the effectiveness of such changes is slim. This study summarizes the literature on recent efforts in the United States to change food-related policies to prevent obesity and diabetes among adults.

**Methods:**

We conducted a systematic review of evidence of the impact of food policies. Websites of government, academic, and nonprofit organizations were scanned to generate a typology of food-related policies, which we classified into 18 categories. A key-word search and a search of policy reports identified empirical evaluation studies of these categories. Analyses were limited to strategies with 10 or more reports. Of 422 articles identified, 94 met these criteria. Using publication date, study design, study quality, and dietary outcomes assessed, we evaluated the strength of evidence for each strategy in 3 assessment categories: time period, quality, and study design.

**Results:**

Five strategies yielded 10 or more reports. Only 2 of the 5 strategies, menu labeling and taxes on unhealthy foods, had 50% or more studies with positive findings in at least 2 of 3 assessment categories. Most studies used methods that were rated medium quality. Although the number of published studies increased over 11 years, study quality did not show any clear trend nor did it vary by strategy.

**Conclusion:**

Researchers and policy makers can improve the quality and rigor of policy evaluations to synthesize existing evidence and develop better methods for gleaning policy guidance from the ample but imperfect data available.

## Introduction

According to the US Centers for Disease Control and Prevention (CDC), more than 29 million Americans have diabetes and 86 million more have prediabetes (1). The International Diabetes Foundation predicts that by 2030, 552 million people worldwide will have diabetes, an increase of more than 50% from 2011 (2). The costs that diabetes imposes in premature death, avoidable comorbidities, and medical expenses make a compelling case for more attention to primary and secondary prevention. Strong evidence suggests that improvements in diet and physical activity can substantially reduce the risk of type 2 diabetes, slow disease progression, and prevent complications among those diagnosed with the disease (3,4).

Any comprehensive diabetes prevention strategy must include policy as well as clinical and community components (5–7). However, although researchers and public health professionals agree on this point in principle, simply implementing more policies does not establish an effective and efficient foundation for diabetes prevention. Thus, policy makers and researchers call for “evidence-based policy,” defined as policies that use data to define objectives, strategies, and outcomes (8). This demand cuts across issues and sectors, including medicine, education, and law enforcement (9–12). There is also a demand for evidence to guide policy related to dietary improvement and diabetes prevention (13). The rationale for evidence-based policy is that it can improve effectiveness and reduce costs while enhancing the credibility of public services and policies (14,15).

In practice, however, the appeal for more emphasis on diabetes prevention policy and more evidence on which to base such policies presents policy makers, researchers, and advocates with a dilemma. On the one hand, epidemiological evidence shows that dietary changes during the past few decades have contributed to increases in diabetes incidence. Growth in overall caloric intake and calories from added sugar indicate that efforts to reverse these dietary trends are needed (16). On the other hand, the recent focus on developing policies to influence diet and the challenges of evaluating their impact have meant that the evidence on which to base recommendations is often slim.

This dilemma presents 2 less than satisfactory choices. Policy makers can wait for the evidence, often delaying action for years and thus failing to prevent premature death or avoidable illness. Or, they can weigh the existing incomplete evidence, making policy decisions based on uncertainty that could fail to generate needed support from other policy makers and the general public. Although acting on persuasive but incomplete evidence constitutes a core public health value (17), policies without strong evidence may incur the opposition of special interest groups (eg, the food industry) or yield unanticipated consequences. Of course, evidence is only one of many factors, including power, advocacy, and special interests, that influence policy decisions. However, for health professionals and researchers, producing more policy-relevant evidence could contribute to policy decisions that improve health. Many policy-making decisions are made without the benefit of strong evidence, yet these same policies are generating natural experiments that will contribute to our growing evidence base.

Our review addresses this dilemma by examining existing evidence on policy initiatives aimed at improving dietary behaviors and reducing or preventing obesity and diabetes in the United States, building on recent reviews that assess evidence on policy interventions in Europe (18,19) and low- and middle-income nations (20). Because our aim was to capture a sense of the full breadth of research in this area, we defined policy as both formal laws or regulations and guidelines and recommendations aimed at supporting healthier behaviors. We also expanded our scope to include any policies with the potential to influence diet, even if they did not directly aim to prevent or reduce obesity and diabetes (eg, international trade policies). To help researchers and policy makers develop more effective approaches to assess whether food and dietary policies can help prevent diabetes and other diet-related chronic diseases, our review asked the following questions:

1. What are the characteristics of studies on diet-related policies, including the strategies they examine, period of publication, study design, and study quality? Has this distribution changed over time?

2. What is the quality of evidence in these studies, and how does it differ by strategy?

3. What is the strength of evidence that specific strategies can lead to changes associated with the prevention of diabetes? Is there more evidence for some strategies than others?

4. How can we strengthen the existing evidence base to advance effective policy making?

## Methods

### Data sources

In 2 phases, we conducted a systematic search for evidence of the impact of policies related to food and nutrition. In the first, we scanned the websites of relevant government, academic, policy, health, and nonprofit organizations to generate a typology of food-related policies and initiatives.

In the second phase, we used this typology to conduct a key-word search across PubMed, Cumulative Index to Nursing and Allied Health Literature (CINAHL) and Web of Science for English-language peer-reviewed scientific, public policy, and practice literature, and a more general search of “gray literature” that included government and nongovernmental agency reports to identify evaluation studies of food-related policies. We cross-referenced health and nutrition key words (eg, body mass index [BMI], diabetes, and obesity) with policy key words (eg, efficacy, intervention, and food access). Throughout this iterative process, our review often brought us to related websites and publications, which led us to identify additional policies and initiatives and add them to our typology. A full list of websites and key words used in each phase is in Appendix A.

To focus our analysis, we limited our review to studies of food-related policies published from January 2000 through December 2011, a time that can be characterized as a “second generation” of renewed interest in food policy. The first-generation studies of the 1990s focused on understanding the scope and causes of growing epidemics of obesity and diet-related disease (21). The second generation of research examined the effectiveness of existing policy approaches and the potential of new ones, such as the regulations on menu calorie labeling implemented in the United States during 2007 and 2008 (22). This analysis can help to inform and influence the robust third generation of food policy efforts currently under way.

### Study selection

We limited our review to studies conducted in the United States, based on the premise that differences in governance and culture might yield different results from similar policies in different nations. We included only studies that focused on adults aged 18 or older, because other researchers have reviewed the literature of obesity and diabetes prevention in children (much of which is focused on school-based policies) (23–25), and because improving population health in the coming decade will require obesity prevention for adults as well as children.

In the first phase of our review, we identified many policy strategies that we classified into 18 distinct categories outcomes (Box). This list is similar to an independently generated typology of food policy strategies developed in a review of European food policies (26). To create a conceptual framework for analyzing these 18 policy strategies, we generated 4 main categories of strategies based on the types of problems that the policies professed to address:

**Box. Categories of Food Policies and Strategies to Reduce Diet-Related Disease**

**Food and Nutrition Information**

- Calorie/menu labeling in restaurants
- Food labeling regulations
- Food marketing regulations
- Nutrition guidelines/education/health media campaigns (including breastfeeding)

**Food Availability and Accessibility**

- Provision of food subsidies/ changing food prices
- Improving retail food access (creating new food stores; improving existing stores)
- Commodity speculation
- Agricultural subsidies
- Farmers’ markets
- Community gardens

**Limiting the Availability of Unhealthy Food**

- Taxes
- Agricultural policies
- International trade policies
- Prohibition/regulation of unhealthy foods
- Regulation of advertising/marketing
- Industry self-regulation
- Zoning laws

**Legal Mechanisms** (Tort law)

1. People lack information to make healthy food choices.

2. People lack access to healthy foods.

3. Unhealthy foods are more accessible, cheaper, and better promoted than healthy foods.

4. People and governments lack legal rights or mechanisms for addressing food-related problems.

We then assigned each policy strategy to at least 1 of the 4 problem-based categories. Some strategies were related to more than one category (eg, an agricultural policy such as subsidizing certain crops might affect both the availability of healthy food and of unhealthy food) (Box). These policy strategies were assigned to both categories.

We limited our review to studies that provided evidence on policy impact, rather than articles that presented commentaries, policy analyses, and editorials. We identified 422 articles that met our initial search criteria. Of these, 139 (33%) articles related to the first category (food and nutrition information); 192 (45%) to improving food availability and accessibility; 110 (26%) to limiting unhealthy food availability; 20 (5%) to legal mechanisms; 39 (9%) related to more than 1 category. Only 129 of the 422 articles (31%) reported empirical evidence (ie, data on outcomes of policy).

Because our goal was to synthesize evidence from multiple studies to assess the weight of the evidence on each strategy, we limited our subsequent analyses to those strategies for which we found 10 or more separate reports with empirical findings, a number that increases the confidence of generalizability across jurisdictions. Selected studies also addressed at least 1 of 3 outcome measures most commonly targeted by nutrition policies: food purchasing (which is frequently used as a proxy for consumption), reported or measured food consumption, or body weight/BMI. The 5 strategies that met the 10-study bar (Table 1) related to 3 of the 4 categories. Of the 422 articles we initially identified, 247 (59%) addressed these strategies (Table 1). Ninety-four (38%) of those included empirical findings on 1 or more of the outcomes of interest. Full citations are in Appendix B.

**Table 1:** Categories of Food Policy to Reduce Obesity and Diabetes Among Adults in the United States Addressed in Evaluation Studies, 2000–2011

Problem Statement	Policy Goal	Strategies for Attaining Goals	No. of Articles	Empirical Findings, N (%)
1. Policies that influence food and nutrition information				
People lack information to make healthy food choices	Provide complete and accurate food and nutrition information to consumers in restaurants	A. Calorie/menu labeling in restaurants	62	20 (32)
	Provide complete and accurate food and nutrition information to consumers on packaged food	B. Food labeling regulations	46	19 (41)
2. Policies that influence food availability and accessibility				
People lack access to healthy foods	Change prices of food to promote healthy eating	A. Provision of food subsidies	51	28 (55)
	Change availability of food to promote healthy eating	B. Creation of new food stores and/or improvement of existing food stores	52	13 (25)
3. Policies that limit availability of unhealthy food				
Unhealthy foods are more accessible, cheaper, and better promoted than healthy foods	Make unhealthy foods less accessible and/or less affordable	Taxes on unhealthy foods	36	14 (39)
Total			247	94 (38)

### Data extraction

To assess the evidence base for these 5 strategies, we recorded the following variables for each of our 94 studies: date of publication, study design, study quality (low, medium, or high), and outcomes assessed (purchasing behavior, consumption behavior, or body weight/BMI).

Because of the heterogeneity of the study designs and outcome measures in our sample, no single quality standard could be applied universally to all the studies. After reviewing several different quality rating methods, we chose an adaptation of the Grading of Recommendations, Assessment, Development and Evaluation (GRADE) system (27,28). The GRADE system has been adopted by many organizations to rate quality of evidence and recommendations, including the US Agency for Healthcare Research and Quality and the World Health Organization. We adapted a version that has been used to rate quality of evidence for public health policies (29).

Our quality assessment assigned 10 possible points based on 4 criteria: study design (whether the study was longitudinal and included an appropriately matched control or comparison group), study population (whether the study described population characteristics using objective or well-validated self-report measures, as well as representativeness and response rate, and included a large [>500] sample size), the description of a specific policy or intervention, and the use of appropriate analytic techniques, including outcomes, statistics, and description of limitations (Appendix C). Studies that scored 0 to 5.0 were considered “low” quality; those scoring 5.5 to 8.5 were considered “medium” quality, and those scoring 9.0 or 10.0 were considered “high” quality. To ensure reliability of our rating system, one researcher coded all 94 studies, and 2 additional researchers independently coded a random 20% subsample. Discrepancies were found in fewer than 6% of indicators across the subsample. The 3 coders met to discuss these discrepancies, and once all 3 reached consensus, the results allowed us to further clarify quality criteria. For instance, we chose to award partial points in the “longitudinal” category to studies using pre–post measures with no control group, which may provide higher-quality results than one-time measures alone.

Finally, we used data from these analyses as the basis for determining the overall strength of evidence for each policy approach. Because no one standard exists for evaluating a heterogeneous group of policy studies, we determined the strength and consistency of evidence by examining the proportion of positive (ie, policy contributed to stated goals), negative (ie, policy was detrimental to stated goals), or mixed (some positive, some negative or null) findings across our 3 main assessment categories: year of publication, study design, and study quality. This approach is similar to that of other systematic reviews that have attempted to capture a breadth of study designs (19,30). We considered studies published in the most recent time frame (2008–2011) to be most applicable to current policy and experimental studies and quasi-natural experiments to be stronger than observational ones. In policy research, quasi-experimental studies or natural experiments, which look at effects across a broad population, may provide stronger evidence than “gold standard” experiments, which look at the behavior of a smaller number of subjects in a controlled environment.

## Results

**Study design.** Of the 94 studies reviewed, 49 (52%) were observational, often using secondary data from nationally representative surveys such as the National Health and Nutrition Examination Survey (NHANES), and 27 (29%) were quasi-experimental or natural experiments. These studies looked at implementation of new policies, such as the introduction of calorie labeling on menus in New York City. Another 8 (9%) were experimental, and 9 (10%) were model estimates, largely assessing effects of taxes on predicted consumption of unhealthy foods and sugar-sweetened beverages. One was a meta-analysis.

**Study quality. **Ten (11%) of the studies in our sample were rated low quality, 67 (71%) medium quality, and 17 (18%) high quality. Quality scores ranged from 3 to 10, and the mean quality score for the full sample (7.1), as well as each individual strategy, was in the medium category (Table 2). Because a large proportion of studies fell into the medium category, we further analyzed this group by “medium-low” (5.5–7.0) and “medium-high” (7.5–8.5) quality scores. Two-thirds of these studies (n = 44) were in the medium-low group, and only 23 were in the medium-high group. The lowest mean quality scores were in the Calorie/Menu Labeling and Creation/Improvement of New Food Stores categories (6.7 and 6.6 respectively), and the highest (7.5) was in the Food Subsidies category, although these differences were not significant.

**Table 2:** Characteristics of Evaluation Studies of Food Policies to Reduce Obesity and Diabetes Among Adults in the United States, 2000–2011

Variable, n (%)	Calorie/Menu Labeling in Restaurants	Food Labeling Regulations	Food Subsidies	Creation or Improvement of Food Stores	Taxes on Unhealthy Foods	All Policies^a^
	n = 20	n = 19	n = 28	n = 13	n = 14	n = 94
**Publication dates**						
2000–2003	0	7 (37)	5 (18)	1 (8)	0	13 (14)
2004–2007	2 (10)	4 (21)	7 (25)	4 (31)	2 (14)	19 (20)
2008–2011	18 (90)	8 (42)	16 (57)	8 (62)	12 (86)	62 (66)
**Study design**						
Observational	5 (24)	14 (74)	16 (57)	10 (77)	4 (29)	49 (52)
Experimental	3 (15)	1 (5)	1 (4)	0	3 (21)	8 (9)
Quasi/natural experiment	11 (55)	4 (21)	7 (25)	3 (23)	2 (14)	27 (29)
Model estimate	1 (5)	0	3 (11)	0	5 (36)	9 (10)
Meta-analysis	0	0	1 (4)	0	0	1 (1)
**Quality ratings**						
Low (0–5.0)	2 (10)	1 (5)	2 (7)	3 (23)	2 (14)	10 (11)
Medium (5.5–8.5)	17 (85)	15 (79)	18 (64)	9 (69)	8 (57)	67 (71)
Medium-low (5.5–7.0)	13 (30)	8 (18)	10 (23)	6 (14)	7 (16)	44 (47)
Medium-high (7.5–8.5)	4 (17)	7 (30)	8 (35)	3 (13)	1 (4)	23 (24)
High (9.0–10.0)	1 (5)	3 (16)	8 (29)	1 (8)	4 (29)	17 (18)
Mean quality score	6.7	7.2	7.5	6.6	7.1	7.1
Mean quality rating	Medium	Medium	Medium	Medium	Medium	Medium
**Dietary and health outcomes assessed**						
Purchase^b^	18 (90)	8 (42)	12 (43)	4 (30)	9 (64)	51 (53)
Consumption	6 (30)	12 (63)	12 (43)	6 (46)	7 (50)	43 (45)
Body weight or body mass index	1 (10)	2 (11)	14 (50)	4 (30)	10 (71)	32 (33)

a Numbers may not sum to total because of rounding.

b Purchase is often used as a proxy for consumption.

**Outcomes assessed.** Of the 94 studies evaluated, 51 (54%) assessed effects on food purchase; 43 (46%) assessed effects on food consumption; and 32 (34%) assessed effects on body weight or composition (Table 2). Of the studies measuring 1 or more outcomes, 37 of 51 (73%) assessing changes in purchasing found a positive effect (ie, the study found a positive association between the policy and the outcome of interest); 29 of 43 (67%) assessing changes in consumption found a positive effect; and 13 of 32 (41%) assessing weight or BMI found a positive effect. One study (2%) assessing changes in consumption and 7 (22%) studies assessing body weight/BMI found negative effects. In addition, 7 (14%) studies assessing changes in purchasing and 7 (16%) assessing changes in consumption found a positive effect, and 4 studies (12%) assessing body weight/BMI found mixed effects. The remaining studies showed no significant effect.

Overall, 61 (65%) of the studies in our sample had positive findings, and only 7 (7%) had negative findings. Twenty studies (21%) had either mixed positive and negative or null findings. Thirteen (65%) of the studies assessing menu labeling had positive findings, while none had negative findings; 14 (74%) assessing packaged food labeling had positive findings, while none had negative findings; 16 studies (57%) addressing food subsidies had positive findings, although 5 (18%) had negative findings; 9 (69%) studies assessing the improvement or creation of food stores had positive findings, while 2 (15%) had negative findings; and 9 (64%) studies on taxing unhealthy foods had positive findings, while none had negative findings.

**Overall strength of evidence.** Overall, 40 studies (43%) in the most recent period showed positive findings, while 6 (6%) showed negative findings. Only 24 studies (26%) with a strong study design showed positive findings, while none showed negative findings. Fifty-four (57%) of our medium- and high-quality studies showed positive findings for any outcome, while 7 (7%) showed negative findings. Across the 5 policy categories, only 2 (calorie/menu labeling and taxing unhealthy foods) found positive results in 50% or more of the studies in at least 2 of our 3 main assessment categories. Most studies with positive findings assessed purchasing behavior. Those assessing body weight and BMI had the fewest studies with positive findings as well as the highest proportion with negative findings (Table 3).

**Table 3:** Strength of Evidence in Studies (n = 94) of Food Policies to Reduce Obesity and Diabetes Among Adults in the United States, 2000–2011

Study Topics	Purchase		Consumption		BMI		Any Outcome	
	Positive^a^	Negative^b^	Positive	Negative	Positive	Negative	Positive	Negative
**Calorie/Menu labeling (n = 20)**								
**All studies**							13 (65)	0
Recent **c**	10 (50)	0	3 (15)	0	1 (5)	0	12 (60)	0
Strong study design **d**	6 (3)	0	3 (15)	0	0 (0)	0	8 (40)	0
Medium or high quality **e**	10 (50)	0	3 (15)	0	1 (5)	0	12 (60)	0
**Food labeling regulations (n = 19)**								
**All studies**							14 (74)	0
Recent^c^	3 (16)	0	5 (26)	0	0	0	7 (37)	0
Strong study design	2 (11)	0	1 (5)	0	1 (5)	0	4 (21)	0
Medium or high quality	5 (26)	0	10 (53)	0	1 (5)	0	14 (74)	0
**Food subsidies (n = 28)**								
**All studies**							16 (57)	5 (18)
Recent^c^	6 (21)	0	5 (18)	1 (4)	1 (4)	4 (14)	9 (32)	4 (14)
Strong study design	5 (18)	0	3 (11)	0	0	0	6 (21)	0
Medium or high quality	10 (36)	0	7 (25)	1 (4)	3 (10)	4 (14)	15 (54)	5 (18)
**Creation or improvement of food stores (n = 13)**								
**All studies**							9 (69)	2 (15)
Recent^c^	3 (23)	0	1 (8)	0	0	2 (15)	4 (31)	2 (15)
Strong study design	3 (23)	0	1 (8)	0	0	0	3 (23)	0
Medium or high quality	2 (15)	0	3 (23)	0	1 (8)	2 (15)	6 (46)	2 (15)
**Taxes on unhealthy foods (n = 14)**								
**All studies**							9 (64)	0
Recent^c^	6 (43)	0	4 (29)	0	6 (43)	0	8 (57)	0
Strong study design	2 (14)	0	1 (7)	0	2 (14)	0	3 (21)	0
Medium or high quality	5 (36)	0	4 (29)	0	1 (8)	0	7 (50)	0
**All studies (n = 94)**								
**All studies**							61 (65)	7 (7)
Recent^c^	28 (30)	0	18 (19)	1 (1)	8 (9)	6 (6)	40(43)	6 (6)
Strong study design	18 (19)	0	9 (10)	0	2 (3)	0	24(26)	0
Medium or high quality	32 (34)	0	27 (29)	1 (1)	12 (13)	7 (8)	54(58)	7 (8)

Abbreviation: BMI, body mass index.

a Positive outcome: significant findings (or for qualitative reports, significant as determined by study authors) that the policy/program contributed to stated goal.

b Negative outcome: significant findings (or for qualitative reports, significant as determined by study authors) that the policy/program was detrimental to stated goal.

c Recent: published 2008–2011.

d Strong study design: experimental/quasi-experimental/natural experiment.

e Medium/high quality: quality rating >5.

## Discussion

Our review found the 10 or more studies we defined as our standard for only 5 of the 18 policy strategies we identified. By this criterion, most dietary policy strategies lack sufficient evidence to determine whether or not they are effective across settings and populations. However, we also found that the number of diet-related policy studies has grown substantially in recent years, a promising trend.

Many studies addressing these 5 policy strategies also employed subjective outcome measures. Consumption and food purchasing were the most frequently used outcomes; the former is typically self-reported, while the latter often uses secondary data analysis of commercial databases, rather than objective physiological measures (eg, BMI, glucose, or hemoglobin Alc levels) or individual purchases over time. Most observers acknowledge the limitations of self-reported dietary information (31). Notably, studies that used biomarkers such as BMI showed the fewest positive and highest negative proportion of results.

Our assessment of the quality of evidence found that most studies were rated medium-quality, largely because they were not longitudinal or did not include an appropriate comparison group other than change over time. Although the number of published studies increased during the 11-year interval reviewed, the quality of studies did not show any clear trend nor did quality vary significantly by strategy. Most studies in our sample were observational, raising questions about the generalizability of their results to real-world policy. These findings suggest the importance of producing more high-quality, rigorous studies of the impact of food policies using standard, validated health outcome measures and, for generalizability, more quasi/natural experimental studies that look at broad populations, particularly in jurisdictions where these strategies have already been implemented.

We found that only 2 of the 5 strategies we identified — menu labeling and taxes on unhealthy foods — had most studies with positive findings for at least 2 of 3 of our main assessment criteria (recently published, strong study design, and medium or high quality). Several factors may explain these results. First, because the interest in policy as a prevention strategy for obesity and diabetes is recent, the body of evidence is only beginning to accumulate. Our finding that the number of published empirical policy evaluation studies increased significantly over the 11 years covered by our review supports this view. Because it may take a decade or more for rigorous evaluation studies to evolve, the recent interest in policy strategies to prevent obesity and diet-related disease suggests that more time may be needed to develop a large and consistent evidence base. Some evidence for this hypothesis is provided by our finding that the mean quality score for studies of food subsidies (7.5), which have been studied for decades, was modestly higher than that for the full sample (7.1). Finally, policy evaluations by nature are complex, and the standards for evaluations that can inform policy are not well established.

Our study had limitations. Our search methods may have missed relevant studies, especially if they appeared only in the “gray literature.” Most systems for rating the quality of evidence have been developed for clinical studies and although the GRADE approach we modified has been used to rate policy studies, the criteria may not adequately assess generalizability and replicability, key indicators for predicting impact on population health. In addition, we found the highest proportion of positive findings in the studies assessing changes in food purchasing, an intermediate outcome that is difficult to tie directly to changes in eating behavior or health status because high proportions of purchased food are wasted (32). The breadth of studies we reviewed did not enable us to look at effect size consistently. By limiting our review to studies published before 2012, we did not include the most recent studies of food policies. However, our synthesis of this generation of policy studies can identify gaps that still need to be filled. Finally, our review does not consider the potential value of the “health-in-all-policies” approach (33) to reducing diet-related diseases because we were unable to identify a sufficient number of studies to assess the benefits of, for example, improving diet by raising the minimum wage or making housing more affordable, nonfood policies that may allow families to spend more on healthier food.

Our review set out to determine whether waiting for evidence on the impact of changes in dietary policy is preferable to weighing the available evidence to inform current policy. We found that neither approach by itself is satisfactory. Waiting for evidence before creating policy interventions may fail to prevent the well-documented consequences of increasing rates of obesity and type 2 diabetes. And simply weighing the inadequate and imperfect existing evaluation studies that we summarize here will not provide a robust guide to more effective policies. Rather, the real question may be how to find the optimal balance between these 2 strategies. This requires taking actions that ensure we can answer the call for evidence-based policy that uses data to define objectives, strategies, and outcomes. To accomplish this, we must increase the quality and quantity of relevant policy evaluation studies and improve our methods for synthesizing available, if imperfect, evidence.

The following strategies may help the field more rapidly accumulate and synthesize evidence:

- Developing standards for specific outcomes and measurement strategies to be used in different types of food policy evaluation studies, including creating and incorporating validated measures and objective biomarkers.
- Using newly implemented and existing policies as an opportunity for natural experiments across broad populations.
- Ensuring that all dietary policy interventions include an adequately funded, rigorously designed evaluation component to assess both implementation and impact.
- Developing methods and models for evaluating the cumulative impact of policy change in dietary behaviors across multiple domains and strategies.
- Studying more systematically and earlier in the policy process the acceptability of new policy solutions to various stakeholder groups.

To achieve the second goal (ie, to better translate the rapidly growing body of food policy evaluation literature into useful information for policy makers), we make the following suggestions:

- Developing standard, user-friendly quality methods to extract policy guidance from evaluation studies. These studies include systematic reviews, thematic syntheses, environmental scans, health impact assessments, and comparative effectiveness reviews — types of evidence that were largely absent in our review.
- Creating national and international workgroups to summarize and widely disseminate findings from evaluation studies of food policies (34).
- Reviewing for relevant lessons how other fields, such as tobacco control (35) and HIV prevention (36), have created and translated evidence into policy guidance.

Policy makers and public health officials have had to ask what constitutes sufficient evidence for making changes. By working simultaneously to improve the quality and rigor of existing and future policy evaluations and to develop new methods to glean policy guidance from the ample but imperfect evidence that does exist, researchers can contribute to policies that lower the burden of premature mortality and preventable illness from diet-related diseases.

## Appendix A Websites and Search Terms

This file is available for download as a Microsoft Word document.

## Appendix B Food Policy Studies Using Empirical Evidence, 2000–2011

This file is available for download as a Microsoft Word document.

## Appendix C Study Quality Assessment Rating Guide

This file is available for download as a Microsoft Word document.
