# Supplementary material for: The State of Evaluation Research on Food Policies to Reduce Obesity and Diabetes Among Adults in the United States, 2000–2011
Source: Prev Chronic Dis. 2015 Oct 29;12:E182. doi: 10.5888/pcd12.150237 (PMC4651114; doi:10.5888/pcd12.150237)
Supplement: Supplementary file 1 [file 15_0237_AppendixA.docx]

**APPENDIX A: Websites and Search Terms Used For Policy and Literature Scan**

1. Initial websites consulted to establish list of food policies and initiatives

American Diabetes Association (www.diabetes.org)

Center for Disease Control and Prevention (www.cdc.gov)

Center for Science in the Public Interest (www.cspinet.org)

Corporations and Health Watch (www.corporationsandhealth.org)

Environmental Working Group (www.ewg.org)

Federal Trade Commission (www.ftc.gov)

Food Politics (www.foodpolitics.com)

Institute for Agriculture and Trade Policy (www.iatp.org)

Institute for Alternative Futures (www.altfutures.com)

Institute of Medicine (www.iom.edu)

International Food Policy Research Institute (www.ifpri.org)

National Conference of State Legislatures (www.ncsl.org)

New York Academy of Sciences (www.nyas.org)

New York City Department of Health and Mental Hygiene (www.nyc.gov/health)

Physicians Committee for Responsible Medicine (www.pcrm.org)

Robert Wood Johnson Foundation (www.rwjf.org)

Rudd Center for Food Policy and Obesity (www.yaleruddcenter.org)

Trust for America’s Health (www.healthyamericans.org)

United States Department of Agriculture (www.usda.gov)

United States Department of Health and Human Services (www.hhs.gov)

United States Food and Drug Administration (www.fda.gov)

1. **Key words**

Search criteria limited to adults 18 and over, English-language, publication between Jan 2000 and December 2011. Policy and health/nutrition keywords were cross-referenced.

**Policy key words**

Access

Advertising

Agriculture

Cause marketing

Community garden; community gardens; community gardening

Efficacy

Environment; environmental

Evidence

Farmers’ market

Health claims

Intervention

Labeling, Posting

Legislation

Menu labeling

Policy

Price

Supermarket, Store, Retail

Subsidy/subsidies

Tax (Food tax; soda tax; food taxation; fat tax; junk food tax; sin tax)

Trade liberalization

Trade policy

**Health and nutrition key words**

BMI, Body Mass Index

Calorie

Diabetes

Diet

Fast food

Food

Functional foods

Hypertension

Nutrition

Overweight; Obesity

Salt

Snack; Snack food

Soda; sugar sweetened beverage; SSB

Sugar

Trans fat
