# Supplementary material for: The State of Evaluation Research on Food Policies to Reduce Obesity and Diabetes Among Adults in the United States, 2000–2011
Source: Prev Chronic Dis. 2015 Oct 29;12:E182. doi: 10.5888/pcd12.150237 (PMC4651114; doi:10.5888/pcd12.150237)
Supplement: Supplementary file 2 [file 15_0237_AppendixB.docx]

***Wait for the Evidence, or Weigh the Evidence?***

**Appendix B: Food Policy Studies using Empirical Evidence, 2000-2011**

**1.A. Calorie/Menu Labeling in Restaurants**

1. Bassett MT et al. Purchasing behavior and calorie information at fast-food chains in New York City 2007. *Am. J. Public Health*, 2008;98:8:1-3.
2. Bleich SN, Pollack KM. [The public’s understanding of daily caloric recommendations and their perceptions of calorie posting in chain restaurants.](http://www.ncbi.nlm.nih.gov/pubmed/20214811)*BMC Public Health.* 2010;10:121.
3. Bollinger B, Leslie P, Sorensen A. *Calorie Posting in Chain Restaurants*, National Bureau of Economic Research Working Paper Series, Vol. w15648. January 2010. Available at: <http://www.nber.org/papers/w15648.pdf>. Accessed August 1, 2014.
4. Burton S et al. Attacking the obesity epidemic: The potential health benefits of providing nutrition information in restaurants.*Am J Public Health*.2006;96:9:1669-1675.
5. Chu YH, Frongillo EA, Jones SJ, Kaye GL. Improving patrons' meal selections through the use of point-of-selection nutrition labels. *Am J Public Health*. 2009; 99(11):2001-5.
6. Dumanovsky T, Huang CY, Bassett MT, Silver LD. Consumer awareness of fast-food calorie information in New York City after implementation of a menu labeling regulation. *Am J Public Health*.2010;100(12):2520-2525.
7. Dumanovsky T, Huang CY, Nonas CA, Matte TD, Bassett MT, Silver LD. Changes in energy content of lunchtime purchases from fast food restaurants after introduction of calorie labeling: cross sectional customer surveys. *BMJ*. 2011;26;343:d4464. doi: 10.1136/bmj.d4464.
8. Elbel B, Kersh R, Brescoll VL, Dixon LB. Calorie Labeling and Food Choices: A First Look at the Effects on Low-Income People in New York City. *Health Aff (Millwood)*. 2009;28(6):w1110-1121.
9. Finkelstein EA, Strombotne KL, Chan NL, Krieger J. [Mandatory menu labeling in one fast-food chain in King County, Washington.](http://www.ncbi.nlm.nih.gov.proxy.wexler.hunter.cuny.edu/pubmed/21238859)*Am J Prev Med*. 2011;40(2):122-7.
10. Freedman MR. Point-of-Selection Nutrition Information Influences Choice of Portion Size in an All-You-Can-Eat University Dining Hall. *J Foodservice Business Research*2011;14(1):86-98.
11. [Gerend MA](http://www.ncbi.nlm.nih.gov.proxy.wexler.hunter.cuny.edu/pubmed?term=%22Gerend%20MA%22%5BAuthor%5D). Does calorie information promote lower calorie fast food choices among college students? *J Adolesc Health*. 2009;44(1):84-6.
12. Harnack LJ, French SA, Oakes JM, Story MT, Jeffery RW, Rydell SA. Effects of calorie labeling and value size pricing on fast food meal choices: Results from an experimental trial. *Int J BehavNutrPhys Act.*2008;5:63.
13. Krukowski, RA, Harvey-Berino, J, Narsana RT, DeSisto TP. Consumers May Not Use or Understand Calorie Labeling in Restaurants. *J Am Diet Assoc*. 2006;106(6):917–20.
14. Kuo T, Jarosz CJ, Simon P, Fielding JE. Menu labeling as a potential strategy for combating the obesity epidemic: A health impact assessment. *Am J Public Health,*2009;99(9):1680-1686.
15. Lowe MR, Tappe KA, Butryn ML, Annunziato RA, Coletta MC, Ochner CN et al. An intervention study targeting energy and nutrient intake in worksite cafeterias. *Eating Behaviors*.2010;11(3):144–151.
16. [Piron J](http://www.ncbi.nlm.nih.gov.proxy.wexler.hunter.cuny.edu/pubmed?term=%22Piron%20J%22%5BAuthor%5D), [Smith LV](http://www.ncbi.nlm.nih.gov.proxy.wexler.hunter.cuny.edu/pubmed?term=%22Smith%20LV%22%5BAuthor%5D), [Simon P](http://www.ncbi.nlm.nih.gov.proxy.wexler.hunter.cuny.edu/pubmed?term=%22Simon%20P%22%5BAuthor%5D), [Cummings PL](http://www.ncbi.nlm.nih.gov.proxy.wexler.hunter.cuny.edu/pubmed?term=%22Cummings%20PL%22%5BAuthor%5D), [Kuo T](http://www.ncbi.nlm.nih.gov.proxy.wexler.hunter.cuny.edu/pubmed?term=%22Kuo%20T%22%5BAuthor%5D). Knowledge, attitudes and potential response to menu labeling in an urban public health clinic population. *Public Health Nutr*. 2010;13(4):550-5.
17. Pulos E, Leng K. Evaluation of a voluntary menu-labeling program in full-service restaurants. *Am J Public Health*. 2010;100(6):1035-1039.
18. Roberto CA, Larsen PD, Agnew H, Baik J, Brownell KD. Evaluating the impact of menu labeling on food choices. *Am J Public Health.* 2010;100(2):312-318.
19. Vadiveloo MK, Dixon LB, Elbel B. Consumer purchasing patterns in response to calorie labeling legislation in New York City. *Int J BehavNutr Phys Act*. 2011;27;8(1):51.
20. Wisdom J, Downs JS, Loewenstein G. Promoting healthy choices: information versus convenience.*American Economic Journal: Applied Economics*, 2010:2(2):164-178.

**1.B. Food Labeling Regulations**

1. Drichoutis AC, Nayga RM, Lazaridis P. Can nutritional label use influence body weight outcomes? *Kyklos.* 2009*:*62(4):500-525.
2. Fitzgerald N, Damio G, Segura-Perez S, Perez-Escamilla R. Nutrition knowledge, food label use, and food intake patterns among Latinas with and without type 2 diabetes. *J Am Diet Assoc*, 2008;108(6):960-967.
3. Lin CT, Lee JY, Yen ST. Do dietary intakes affect search for nutrient information on food labels?*Social Science & Medicine*, 2004;59(9):1955-1967.
4. Kim SY, Nayga RM, Capps OR. The effect of food label use on nutrient intakes: an endogenous switching regression analysis*. Journal of Agricultural Economics,*2000;25(1):215–231.
5. Kim SY, Nayga R, Capps A. Food label use, self-selectivity, and diet quality, *Journal of Consumer Affairs*. 2001;35:346–363.
6. Lando AM, Labiner-Wolfe J. Helping Consumers Make More Healthful Food Choices: Consumer Views on Modifying Food Labels and Providing Point-of-Purchase Nutrition Information at Quick-service Restaurants. *Journal of Nutrition Education and Behavior.*2007;39(3):157-63.
7. Lang JE, Mercer N, Tran D, Mosca L. Use of a supermarket shelf labeling program to educate a predominately minority community about foods that promote heart health. *J Am Diet Assoc.* 2000;100: 804–9.
8. Lewis JE, Arheart KL, LeBlanc, WG, Fleming LE, Lee DJ, Davila EP et al. Food label use and awareness of nutritional information and recommendations among persons with chronic disease. *Am J Clinical Nutrition*.2009;90(5):1351-57.
9. McArthur L, Chamberlain V, Howard AB. Behaviors, attitudes, and knowledge of low-income consumers regarding nutrition labels. *J Health Care Poor Underserved.* 2001;12(4):415-28.
10. Niederdeppe J, Frosch DL. [News coverage and sales of products with transfat: effects before and after changes in federal labeling policy.](http://www.ncbi.nlm.nih.gov.proxy.wexler.hunter.cuny.edu/pubmed/19269126)*Am J Prev Med*. 2009;36(5):395-401.
11. Ollberding NJ, Wolf RL, Contento I. Food label use and its relation to dietary intake among US adults. *J Am Diet Assoc*. 2010;110(8):1233-7.
12. Pérez-Escamilla R, Haldeman L. Food label use modifies association of income with dietary quality. *Journal of Nutrition.*2002;132(4):768-772.
13. Post RE, Mainous AG, Diaz V, Matheson, EM, Everett, CJ. Use of the nutrition facts label in chronic disease management: Results from the National Health and Nutrition Examination Survey. *J Am Diet Assoc*.2010;110(4):628-632.
14. Satia JA, Galanko JA, Neuhouser ML. Food nutrition label use is associated with demographic, behavioral, and psychosocial factors and dietary intake among African Americans in North Carolina. *J Am Diet Assoc*. 2005;105(3):392-402;discussion 402-3.
15. Teisl MF, Bockstael NE, Levy AS. Measuring the welfare effects of nutrition information. *Am JAgricultural Economics*. 2001;83:133–149.
16. Todd JE, Variyam JN. *The Decline in Consumer Use of Food Nutrition Labels, 1995-2006*. ERR-63, USDA, Economic Research Service. August 2008. Available at: http://www.ers.usda.gov/publications/err63/. Accessed August 1, 2014.
17. Variyam JN. Do Nutrition Labels Improve Dietary Outcomes?*Health Economics*.2008;17:695-708.
18. Variyam JN, Cawley J. *Nutrition labels and obesity.* NBER working paper 11956. Cambridge, MA: National Bureau of Economic Research*.* 2006. Available at:www.nber.org/papers/w11956.pdf. Accessed August 1, 2014.
19. Weaver D, Finke M. The relationship between the use of sugar content information on nutrition labels and the consumption of added sugars.*Food Policy*.2003;28(3):213-219.

**2.A. Provision of Food Subsidies**

1. Alston JM, Mullally CC, Sumner DA, Townsend M, Vosti SA. Likely effects on obesity from proposed changes to the US Food Stamp Program. *Food Policy*.2009;34:176-184.
2. [Andreyeva T](http://www.ncbi.nlm.nih.gov.proxy.wexler.hunter.cuny.edu/pubmed?term=%22Andreyeva%20T%22%5BAuthor%5D), [Blumenthal DM](http://www.ncbi.nlm.nih.gov.proxy.wexler.hunter.cuny.edu/pubmed?term=%22Blumenthal%20DM%22%5BAuthor%5D), [Schwartz MB](http://www.ncbi.nlm.nih.gov.proxy.wexler.hunter.cuny.edu/pubmed?term=%22Schwartz%20MB%22%5BAuthor%5D), [Long MW](http://www.ncbi.nlm.nih.gov.proxy.wexler.hunter.cuny.edu/pubmed?term=%22Long%20MW%22%5BAuthor%5D), [Brownell KD](http://www.ncbi.nlm.nih.gov.proxy.wexler.hunter.cuny.edu/pubmed?term=%22Brownell%20KD%22%5BAuthor%5D). Availability and prices of foods across stores and neighborhoods: the case of New Haven, Connecticut. *Health Aff (Millwood).* 2008;27(5):1381-8.
3. Andreyeva T, Middleton AE, Long MW, Luedicke J, Schwartz MB. Food retailer practices, attitudes and beliefs about the supply of healthy foods. *Public Health Nutrition.* 2011;14(6):1024-1031.
4. Baum, CL. The effects of food stamps on obesity. *Southern Economic Journal*.2011;77(3) 623-51.
5. Beydoun MA, Powell LM, Wang Y. The association of fast food, fruit and vegetable prices with dietary intakes among US adults: is there modification by family income? *SocSci Med*. 2008;66(11):2218-29.
6. Chen Z, Yen ST, Eastwood DB. Effects of food stamp participation on body weight and obesity. *American Journal of Agriculture Economy*, 2005;87(5):1167–1173.
7. Dong D, Leibtag E. *Promoting Fruit and Vegetable Consumption: Are Coupons More Effective Than Pure Price Discounts*? ERR-96, USDA, Economic Research Service. June 2010. Available at: <http://www.ers.usda.gov/Publications/ERR96/>. Accessed August 1, 2014.
8. Dong D, Lin BH. *Fruit and Vegetable Consumption by Low-Income Americans: Would a Price Reduction Make a Difference?* Economic Research Report No. (ERR-70). January 2009. Available at:http://www.ers.usda.gov/Publications/err70/. Accessed August 1, 2014.
9. Faith MS, Fontaine KR, Baskin ML, Allison DB. Toward the reduction of population obesity: macrolevel environmental approaches to the problems of food, eating, and obesity. *Psychol Bull*. 2007;133(2):205-26.
10. French SA, Harnack LJ, Hannan PJ, Mitchell NR, Gerlach AF, Toomey TL. Worksite environment intervention to prevent obesity among metropolitan transit workers.*Prev Med*. 2010;50(4):180-5. doi: 10.1016/j.ypmed.2010.01.002.
11. French SA, Jeffery RW, Story M, Breitlow KK, Baxter JS, Hannan P, Snyder MP. Pricing and promotion effects on low-fat vending snack purchases: the CHIPS Study. *Am J Public Health*. 2001 Jan;91(1):112-7.
12. Gibson D. Food stamp program participation is positively related to obesity in low income women. *J Nutr*. 2003;133(7):2225-31.
13. [Herman DR](http://www.ncbi.nlm.nih.gov.proxy.wexler.hunter.cuny.edu/pubmed?term=%22Herman%20DR%22%5BAuthor%5D), [Harrison GG](http://www.ncbi.nlm.nih.gov.proxy.wexler.hunter.cuny.edu/pubmed?term=%22Harrison%20GG%22%5BAuthor%5D), [Afifi AA](http://www.ncbi.nlm.nih.gov.proxy.wexler.hunter.cuny.edu/pubmed?term=%22Afifi%20AA%22%5BAuthor%5D), [Jenks E](http://www.ncbi.nlm.nih.gov.proxy.wexler.hunter.cuny.edu/pubmed?term=%22Jenks%20E%22%5BAuthor%5D). Effect of a targeted subsidy on intake of fruits and vegetables among low-income women in the Special Supplemental Nutrition Program for Women, Infants, and Children. *Am J Public Health*. 2008;98(1):98-105.
14. Herman DR, Harrison GG, Jenks E. Choices made by low-income women provided with an economic supplement for fresh fruit and vegetable purchase. *J Am Diet Assoc.* 2006;106:740-4.
15. Horgen KB, Brownell KD. Comparison of price change and health message interventions in promoting healthy food choices. *Health Psychology*.2002;21(5):505–512.
16. Jilcott SB, Liu H, DuBose KD, Chen S, Kranz S. Food stamp participation is associated with fewer meals away from home, yet higher body mass index and waist circumference in a nationally representative sample. *J NutrEducBehav*. 2011;43:110-115.
17. Kaushal N. Do food stamps cause obesity? Evidence from the immigrant experience. *J Health Economics*2007;26:968–991.
18. Kim K, Frongillo E. Participation in food assistance programs modifies the relation of food insecurity with weight and depression in elders.*J Nutr.*2007;137(4):1005-1010.
19. [Kunkel ME](http://www.ncbi.nlm.nih.gov.proxy.wexler.hunter.cuny.edu/pubmed?term=%22Kunkel%20ME%22%5BAuthor%5D), [Luccia B](http://www.ncbi.nlm.nih.gov.proxy.wexler.hunter.cuny.edu/pubmed?term=%22Luccia%20B%22%5BAuthor%5D), [Moore AC](http://www.ncbi.nlm.nih.gov.proxy.wexler.hunter.cuny.edu/pubmed?term=%22Moore%20AC%22%5BAuthor%5D). Evaluation of the South Carolina seniors farmers' market nutrition education program. *J Am Diet Assoc*. 2003;103(7):880-3.
20. Leung CW, Villamor E. Is participation in food and income assistance programmes associated with obesity in California adults? Results from a state-wide survey. *Public Health Nutr*. 2011;14(4):645-52.
21. Meyerhoefer C, Pylypchuk Y. Does participation in the food stamp program affect the prevalence of obesity and health care spending? *Am J Agricultural Economics*.2008;90(2), 287-305.
22. Powell LM, Zhao Z, Wang Y. Food prices and fruit and vegetable consumption among young American adults. *Health Place*. 2009;15(4):1064-70.
23. Racine EF, Smith Vaughn A, Laditka SB. Farmers' market use among African-American women participating in the Special Supplemental Nutrition Program for Women, Infants, and Children. *J Am Diet Assoc,* 2010;110:441-6.
24. VerPloeg M, Mancino L, Lin B, Wang C. The vanishing weight gap: trends in obesity among adult food stamp participants (US) (1976–2002). *J Nutr.*2007;137:1005–1010.
25. Ver Ploeg M, Mancino L, Lin BH. Food stamps and obesity: Ironic twist or complex puzzle? *Amber Waves*.2006;4(1):32-37. Available at: <http://www.ers.usda.gov/Amberwaves/February06/Features/Feature4.htm>. Accessed August 1, 2014.
26. Webb AL et al. Food Stamp Program participation but not food insecurity is associated with higher adult BMI in Massachusetts residents living in low-income neighbourhoods. *Public health nutrition*2008;11(12):1248-1255.
27. Wilde E, McNamara PE, Ranney CK. *The Effect on Dietary Quality of Participation in the Food Stamp and WIC Programs.* Food Assistance and Nutrition Research Report No. (FANRR9). September 2000. Available at: <http://webarchives.cdlib.org/sw1bc3ts3z/http://ers.usda.gov/publications/fanrr9/>. Accessed July 27, 2014.
28. Zagorsky JL, Smith PK. Does the US Food Stamp Program contribute to adult weight gain? *Econ Hum Biol*. 2009;7(2):246-58.

**2.B. Creation/Improvement of Food Stores**

1. [Bodor JN](http://www.ncbi.nlm.nih.gov/pubmed?term=Bodor%20JN%5BAuthor%5D&cauthor=true&cauthor_uid=17617930), [Rose D](http://www.ncbi.nlm.nih.gov/pubmed?term=Rose%20D%5BAuthor%5D&cauthor=true&cauthor_uid=17617930), [Farley TA](http://www.ncbi.nlm.nih.gov/pubmed?term=Farley%20TA%5BAuthor%5D&cauthor=true&cauthor_uid=17617930), [Swalm C](http://www.ncbi.nlm.nih.gov/pubmed?term=Swalm%20C%5BAuthor%5D&cauthor=true&cauthor_uid=17617930), [Scott SK](http://www.ncbi.nlm.nih.gov/pubmed?term=Scott%20SK%5BAuthor%5D&cauthor=true&cauthor_uid=17617930). Neighbourhood fruit and vegetable availability and consumption: the role of small food stores in an urban environment. [*Public Health Nutr*.](http://www.ncbi.nlm.nih.gov/pubmed/17617930) 2008;11(4):413-20.
2. Bodor JN, Ulmer VM, Futrell Dunaway L, et al. The Rationale behind Small Food Store Interventions in Low-Income Urban Neighborhoods: Insights from New Orleans. *J Nutr.* 2010;140:1185-1188.
3. Boone-Heinonen J, Gordon-Larsen P, Kiefe CI, Shikany JM, Lewis CE, Popkin BM. Fast Food Restaurants and Food Stores: Longitudinal Associations With Diet in Young to Middle-aged Adults: The CARDIA Study. *Arch Intern Med*. 2011;171(13):1162-70.
4. [Casagrande SS](http://www.ncbi.nlm.nih.gov.proxy.wexler.hunter.cuny.edu/pubmed?term=%22Casagrande%20SS%22%5BAuthor%5D), [Franco M](http://www.ncbi.nlm.nih.gov.proxy.wexler.hunter.cuny.edu/pubmed?term=%22Franco%20M%22%5BAuthor%5D), [Gittelsohn J](http://www.ncbi.nlm.nih.gov.proxy.wexler.hunter.cuny.edu/pubmed?term=%22Gittelsohn%20J%22%5BAuthor%5D), [Zonderman AB](http://www.ncbi.nlm.nih.gov.proxy.wexler.hunter.cuny.edu/pubmed?term=%22Zonderman%20AB%22%5BAuthor%5D), [Evans MK](http://www.ncbi.nlm.nih.gov.proxy.wexler.hunter.cuny.edu/pubmed?term=%22Evans%20MK%22%5BAuthor%5D), [FanelliKuczmarski M](http://www.ncbi.nlm.nih.gov.proxy.wexler.hunter.cuny.edu/pubmed?term=%22Fanelli%20Kuczmarski%20M%22%5BAuthor%5D), [Gary-Webb TL](http://www.ncbi.nlm.nih.gov.proxy.wexler.hunter.cuny.edu/pubmed?term=%22Gary-Webb%20TL%22%5BAuthor%5D). Healthy food availability and the association with BMI in Baltimore, Maryland. *Public Health Nutr*. 2011;14(6):1001-7.
5. Courtemanche C, Carden A. Supersizing supercenters? The impact of Wal-Mart supercenters on body mass index and obesity. *J Urban Econ.*2011;69 (2):165–181.
6. Jetter KM, Cassady DL. Increasing fresh fruit and vegetable availability in a low-income neighborhood convenience store: A pilot study. *Health PromotPract*. 2010;11(5):694-702.
7. Laraia BA, Siega-Riz AM, Kaufman JS, Jones SJ. Proximity of supermarkets is positively associated with diet quality index for pregnancy. *Prev Med.* 2004;39:869–75.
8. Morland KB, Evenson, KR. Obesity prevalence and the local food environment. *Health & Place*.2009;15(2):491-495.
9. [Morland K](http://www.ncbi.nlm.nih.gov.proxy.wexler.hunter.cuny.edu/pubmed?term=%22Morland%20K%22%5BAuthor%5D), [Diez Roux AV](http://www.ncbi.nlm.nih.gov.proxy.wexler.hunter.cuny.edu/pubmed?term=%22Diez%20Roux%20AV%22%5BAuthor%5D), [Wing S](http://www.ncbi.nlm.nih.gov.proxy.wexler.hunter.cuny.edu/pubmed?term=%22Wing%20S%22%5BAuthor%5D). Supermarkets, other food stores, and obesity: the atherosclerosis risk in communities study. *Am J Prev Med*. 2006;30(4):333-9.
10. [Morland K](http://www.ncbi.nlm.nih.gov/pubmed?term=Morland%20K%5BAuthor%5D&cauthor=true&cauthor_uid=11777675), [Wing S](http://www.ncbi.nlm.nih.gov/pubmed?term=Wing%20S%5BAuthor%5D&cauthor=true&cauthor_uid=11777675), [Diez Roux A](http://www.ncbi.nlm.nih.gov/pubmed?term=Diez%20Roux%20A%5BAuthor%5D&cauthor=true&cauthor_uid=11777675), [Poole C](http://www.ncbi.nlm.nih.gov/pubmed?term=Poole%20C%5BAuthor%5D&cauthor=true&cauthor_uid=11777675). Neighborhood characteristics associated with the location of food stores and food service places. [*Am J Prev Med*.](http://www.ncbi.nlm.nih.gov/pubmed/11777675)2002;22(1):23-9.
11. [Rose D](http://www.ncbi.nlm.nih.gov/pubmed?term=Rose%20D%5BAuthor%5D&cauthor=true&cauthor_uid=15548347), [Richards R](http://www.ncbi.nlm.nih.gov/pubmed?term=Richards%20R%5BAuthor%5D&cauthor=true&cauthor_uid=15548347). Food store access and household fruit and vegetable use among participants in the US Food Stamp Program. [*Public Health Nutr*.](http://www.ncbi.nlm.nih.gov/pubmed/15548347) 2004;7(8):1081-8.
12. Song HJ, Gittelsohn J, Kim M, Suratkar S, Sharma S, Anliker J. A corner store intervention in a low-income urban community is associated with increased availability and sales of some healthy foods. *Public Health Nutr.* 2009;12:2060-7
13. [Vásquez VB](http://www.ncbi.nlm.nih.gov/pubmed?term=V%C3%A1squez%20VB%5BAuthor%5D&cauthor=true&cauthor_uid=17728199), [Lanza D](http://www.ncbi.nlm.nih.gov/pubmed?term=Lanza%20D%5BAuthor%5D&cauthor=true&cauthor_uid=17728199), [Hennessey-Lavery S](http://www.ncbi.nlm.nih.gov/pubmed?term=Hennessey-Lavery%20S%5BAuthor%5D&cauthor=true&cauthor_uid=17728199), [Facente S](http://www.ncbi.nlm.nih.gov/pubmed?term=Facente%20S%5BAuthor%5D&cauthor=true&cauthor_uid=17728199), [Halpin HA](http://www.ncbi.nlm.nih.gov/pubmed?term=Halpin%20HA%5BAuthor%5D&cauthor=true&cauthor_uid=17728199), [Minkler M](http://www.ncbi.nlm.nih.gov/pubmed?term=Minkler%20M%5BAuthor%5D&cauthor=true&cauthor_uid=17728199). Addressing food security through public policy action in a community-based participatory research partnership. [*Health Promot Pract*.](http://www.ncbi.nlm.nih.gov/pubmed?term=Breckwich%20V%C3%A1squez%20Lanza%202007)2007;8(4):342-9.

**3. Taxes on Unhealthy Foods**

1. Andreyeva T, Chaloupka FJ, Brownell KD. Estimating the potential of taxes on sugar-sweetened beverages to reduce consumption and generate revenue. *Prev Med.* 2011;52(6):413-6.
2. Dharmasena S, Capps O. [Intended and unintended consequences of a proposed national tax on sugar-sweetened beverages to combat the U.S. obesity problem.](http://www.ncbi.nlm.nih.gov.proxy.wexler.hunter.cuny.edu/pubmed/21538676)*Health Econ*. 2012;21(6):669-94. doi: 10.1002/hec.
3. Duffey KJ, Gordon-Larsen P, Shikany JM, Guilkey D, Jacobs DR Jr, Popkin BM. Food price and diet and health outcomes: 20 years of the CARDIA Study. *Arch Intern Med*. 2010;170(5):420-6. Erratum in: *Arch Intern Med*. 2010 Jun28;170(12):1089.
4. Epstein LH, Dearing KK, Roba LG, Finkelstein E. The influence of taxes and subsidies on energy purchased in an experimental purchasing study. *Psychol Sci*. 2010;21(3):406-14.
5. Finkelstein EA, Zhen C, Nonnemaker J, Todd JE. The impact of targeted beverage taxes on higher and lower income households. [*Arch*](http://www.rwjf.org/pr/search.jsp?pubtitle=Archives%20of%20Internal%20Medicine) *Intern Med*, 2010;[170(22)](http://www.rwjf.org/pr/search.jsp?pubtitle=Archives%20of%20Internal%20Medicine&volume=170&number=22):2028-2034.
6. Fletcher JM, Frisvold D, Tefft N. Can soft drink taxes reduce population weight? *Contemporary Economic Policy*.2010;28(1):23-35.
7. Gelbach J, Klick J, Stratmann T. Cheap donuts and expensive broccoli: the effect of relative prices on obesity.SSRN 976484. 2009. Available at: <http://www.law.yale.edu/documents/pdf/Intellectual_Life/JKlick_Cheap_Donuts.pdf>. Accessed August 1, 2014.
8. Giesen JC, Payne CR, Havermans RC, Jansen A. Exploring how calorie information and taxes on high-calorie foods influence lunch decisions. *Am J ClinNutr*. 2011;93(4):689-94.
9. Goldman D, Lakdawalla D, Zeng Y. Food Prices and the Dynamics of Body Weight. NBER Working Paper #15096. National Bureau of Economic Research. June 2009. <http://www.nber.org/papers/w15096.pdf>. Accessed August 1, 2014.
10. Han E, Powell LM. Effect of food prices on the prevalence of obesity among young adults. *Public Health*. 2011;125(3):129-35.
11. Kuchler F, Tegene A, Harris JM. 2004. *Taxing Snack Foods: What to Expect for Diet and Tax Revenues*, AIB-747-08, USDA, Economic Research Service, August 2004. <http://www.ers.usda.gov/Publications/AIB747/aib74708.pdf>. Accessed August 1, 2014.
12. Miljkovic D, Nganje W, deChastenet H. Economic Factors Affecting the Increase in Obesity in the United States: Differential Response to Price.*Food Policy*.2008;33(1):48–60.
13. Smith TA, Lin BH, Lee JY. *Taxing Caloric Sweetened Beverages: Potential Effects on Beverage Consumption,Calorie Intake, and Obesity, ERR-100.* USDA, Economic Research Service. July 2010. Available at: http://www.ers.usda.gov/Publications/ERR100/. Accessed August 1, 2014.
14. Temple JL, Johnson KM, Archer K, Lacarte A, Yi C, Epstein LH. Influence of simplified nutrition labeling and taxation on laboratory energy intake in adults. *Appetite*. 2011;57(1):184-92.
