# Supplementary material for: The State of Evaluation Research on Food Policies to Reduce Obesity and Diabetes Among Adults in the United States, 2000–2011
Source: Prev Chronic Dis. 2015 Oct 29;12:E182. doi: 10.5888/pcd12.150237 (PMC4651114; doi:10.5888/pcd12.150237)
Supplement: Supplementary file 3 [file 15_0237_AppendixC.docx]

| Author |  |
| --- | --- |
| Pub Year |  |
| Study Design   - Observational - Experimental - Quasi/Natural Experiment - Model Estimate - Other | - *Quasi-natural* = difference in two populations not manipulated by researcher |
| Did/would policy affect:  (Positive, Negative, No Effect, Mixed, N/A) | - *Exclude studies that do not address one or more outcomes; exclude studies evaluating process rather than outcome* - *Rate studies with both positive/no effect findings as positive* |
| Purchase | - *Uses data from stores* |
| Consumption | - *Uses observed/reported consumption* |
| BMI/Obesity |  |

**Appendix C: Study Quality Assessment Rating Guide**

| **Quality Measure** | **Quality Scoring** | **Quality Pts.** |
| --- | --- | --- |
| *Design:*  Longitudinal? | Longitudinal = 1  Repeated measures (pre/post with no control) = 0.5  Other = 0 |  |
| *Design:*  Control group? | Appropriately matched control group (comparison group other than change over time) = 1  No/poorly matched group = 0 |  |
| *Population:*  Description | Study describes population characteristics using objective or well-validated self-report measures (e.g., definition of diabetes or pre-diabetes) = 1 |  |
| *Population:* Representativeness | Study describes representativeness of sample to geographic or membership (e.g., of a health plan) population (incomplete studies using secondary data from well-documented representative surveys, e.g. NHANES) = 1 |  |
| *Population:*  Response rate | Study deals with selection by reporting response rate *and* describing responders vs. non-responders (incomplete studies using secondary data from well-documented surveys) = 1 point  Describes either response rate *or* describes responders/non-responders = 0.5 |  |
| *Population:*  Sample size | ≥500 people = 1  <500 people = 0 |  |
| *Policy description* | Study clearly describes components of an existing intervention/policy = 1  Study does not address specific policy, or suggests policy to be implemented at a later time = 0 |  |
| *Analysis:* Outcomes | Appropriate outcome measures = 1 |  |
| *Analysis:* Statistics | Justifies choice of analysis (all studies) and uses appropriate statistical measures (if quantitative)= 1 |  |
| *Analysis:* Limitations | Documents limitations of design, data available, and reasonable interpretations and/or assumptions (for model estimates) = 1 |  |
| **Total quality points** | 1-5 points = low  5.5-8.5 points = moderate  9-10 points = high |  |
